# Post-Traumatic Growth of Nurses in COVID-19 Designated Hospitals in Korea

**DOI:** 10.3390/ijerph20010056

**Published:** 2022-12-21

**Authors:** Suk-Jung Han, Ji-Young Chun, Hye-Jin Bae

**Affiliations:** 1 College of Nursing, Sahmyook University, Seoul 01795, Republic of Korea; 2 Department of Nursing, Seoul Medical Center, Seoul 02053, Republic of Korea

**Keywords:** COVID-19, post-traumatic stress disorder, post-traumatic growth, social support, rumination, resilience

## Abstract

Background: This descriptive survey aimed to identify the factors affecting the post-traumatic growth (PTG) of nurses in COVID-19 designated hospitals on the basis of a PTG model. Methods: A survey of 250 nurses working at three COVID-19 hospitals in Seoul, South Korea, was conducted from May to July 2021. The collected data were analyzed using the IBM SPSS 25 (IBM Inc., Armonk, NY, USA). Results: The participants in this study were mostly women (92.7%), and the average age and career duration were 32.08 and 7.88 years, respectively. The factors that significantly influenced the participants’ PTG were identified as marriage, religion, self-disclosure, deliberate rumination, meaning in life, and resilience. Conclusions: As new infectious diseases emerge, it is necessary to develop a program that can encourage self-disclosure and deliberate rumination, help nurses discover and pursue meaning in life, and enhance their ability to overcome trauma and promote PTG.

## 1. Introduction

With the spread of severe acute respiratory syndrome (SARS) in 2003, the Middle East Respiratory Syndrome (MERS) in 2012, and the COVID-19 infections in 2020 caused by the coronavirus in China, it is evident that the world is still unable to escape the wave of pandemics [1]. According to studies conducted during the COVID-19 pandemic, healthcare workers reported anxiety (15.15%), depression (15.86%), post-traumatic stress disorder (PTSD) (20.91%), insomnia (36.5%), and psychological distress (16.88%) [1,2]. In a meta-analysis of papers published during the SARS, MERS, and COVID-19 pandemics, PTSD was found to be high at 32.2% in the post-disease stage [3]. In particular, the results of a meta-analysis targeting medical staff (doctors, nurses, assistants, etc.) showed that 11–74% showed PTSD, and 10–40% of them showed these symptoms even after 1 to 3 years later [4]. Calhoun and Tedeschi [5] described trauma as an event that disrupts values and beliefs about the existing world and that is so threatening that individuals cannot adapt using existing coping methods; there is a focus on the intensity of individuals’ subjective distress rather than objective threats.

Post-traumatic stress has negative effects on mental and physiological functions caused by exposure to extreme traumatic stress events [6]. It is a symptom in which a person experiences extreme fear or helplessness due to a physical experience, re-experiences a traumatic event continuously for more than one month, avoids stimuli related to trauma, and maintains increased arousal [7]. People who experience trauma suffer pain or maladaptation. However, they can also appreciate life more than before the traumatic event, discover new possibilities and meanings in life, and reset their life goals. Additionally, close relationships become more valuable. As individuals overcome their pain, they develop greater inner strength, spiritual interest, and depth [8]. Post-traumatic growth (PTG) refers to a positive psychological change due to coping with or fighting stressful events [9].

Tedeschi and Calhoun’s PTG model [8] explores the meaning of traumatic events through the individual’s rumination, which facilitates the integration of existing cognitive frameworks and traumatic experiences. Rumination promotes PTG by helping individuals to understand the meaning of the traumatic events and change existing cognitive schemas so the events can be understood.

In the early days of trauma, individuals have automatic and invasive reflections of the traumatic experiences. However, over time, they experience intentional reflections that allow them to find and understand the meaning of events. Simultaneously, they experience self-disclosure by sharing painful experiences with those around them, and thereby receive social support [10,11]. Social support has been reported to lower post-traumatic stress in nurses [12] and is a positive influencing factor of PTG [13]. PTG can develop when receiving social support stably and continuously [14].

PTG can be easily achieved when a traumatized person’s self-disclosure leads to social support and empathy [9]. However, if it doesn’t lead to social support and empathy, this may inflict secondary or tertiary trauma on the victims [15]. In addition, self-disclosure provides emotional comfort and relief; PTG develops through this intentional rumination [14,16].

Having a sense of meaning in life has been defined in various ways, such as having consistency in life, goal orientation or purpose, intention to act, and reason for action [17]. Having meaning in life is also strongly related to mental health and has been reported to have a positive effect on adaptation and coping. People with a sense of meaning in life experience greater happiness, satisfaction, and positive emotions, especially by controlling or mediating the relationship between trauma or stress and mental health, which promotes adaptive coping [18]. It has been reported that having meaning in life serves as a buffer resource that allows one to experience hope for life or PTG even when undergoing adversity or stress [19,20]. Previous studies have reported that having meaning in life positively affects PTG [21,22].

The spread of new infectious diseases worldwide affects everyone, but not everybody responds in the same way [23]. Resilience is the ability to positively recover or grow by challenging environmental crises, endure adversity, and successfully adapt to changes or forces that lead to recovery to the pre-stress level of adaptation [24]. A study of nurses showed that the higher the resilience, the more active the coping with stress and the ability to adapt to stressful situations [25]. Resilience positively predicted PTG [26].

Research on PTG in Korea has mainly targeted cancer patients or general adults who suffered from trauma along with police officers and firefighters [27]. There are a few studies on PTG among nurses [28,29], but no study has yet investigated PTG among nurses working in a hospital designated for COVID-19.

Therefore, this study selected as participants nurses who directly took care of patients in hospitals designated for COVID-19. The general and work-related characteristics of the participants, variables constituting the PTG model, and variables that have been shown to have a positive effect on PTG in previous studies were identified. Based on the results of this study, we intend to provide basic materials for developing a program that promotes PTG.

This study aims to confirm that the components of the post-traumatic growth model of Tedeschi and Calhoun [8] affect post-traumatic growth. It aims to identify the effect of factors such as social support, meaning in life, and resilience, which have been shown to have a positive effect on post-traumatic growth in previous studies on the subject.

The main objective was to determine the factors that affect the PTG of nurses working in hospitals designated for COVID-19, with specific aims to:

1. Identify the degree of PTG according to participants’ demographic characteristics.
2. Identify participants’ post-traumatic event experience, PTSD, self-exposure, social support, rumination, meaning in life, degree of resilience, and PTG.
3. Identify the correlations between PTG and post-traumatic event experience, PTSD, self-exposure, social support, rumination, meaning in life, and resilience.
4. Identify the factors that affect participants’ PTG.

## 2. Materials and Methods

### 2.1. Study Design and Participants

This study was a descriptive survey to explore the factors affecting the PTG of nurses who took care of patients during the COVID-19 pandemic.

The target participants of this study were nurses working at designated hospitals during the COVID-19 pandemic; convenience sampling of 250 nurses from three public hospitals designated as COVID-19 hospitals in Seoul, Republic of Korea, was conducted.

A total of 235 surveys were collected and 233 were analyzed, excluding two copies with some missing responses.

The sample size was calculated using G* Power 3.1.9.2. For multiple regression, with a level of significance of (α) 0.05, power of (1 − β) 0.95, effect size of 0.15 [29], and 18 predictors, the required minimum sample size was calculated to be 213. Considering the dropout rate, a survey was conducted with 250 nurses, and 233 responses were used in the analysis.

### 2.2. Measures

The data were collected through a survey. Regarding the questionnaire items, we chose tools that were tested for reliability and validity in previous studies.

#### 2.2.1. Post-Traumatic Growth

PTG is a positive psychological change that results from combating trauma beyond resisting extreme stress and not being harmed. It includes qualitative changes in the ability to resist trauma or in individual function and adaptation beyond pre-traumatic levels [8]. After Tedeschi and Calhoun [9] developed the PTG Inventory, Tedeschi et al. [30] added existential and spiritual change questions to the scale, and the Post-traumatic Growth Inventory-X (PGTI-X) was developed.

In this study, PTG was assessed using the PTGI-X, which had the validity and reliability of the Korean version, as checked by Kim et al. [27]. This tool consists of 25 items assessing four domains: “change in self-perception” (8 items), “increase in interpersonal depth” (5 items), “increase in spiritual and existential depth” (7 items), and “discovery of new possibilities” (5 items). Participants were asked to identify the degree to which they did or did not experience a particular change (0 *=* I did not experience this change to 5 = I experienced this change to a very great degree). The total PTGI score ranged from 0 to 150, with higher scores indicating greater growth. At the time of development, Cronbach’s α = 0.97 in the study by Kim et al. [27] and was 0.969 in this study.

#### 2.2.2. Experience of the Traumatic Event

Experience of a traumatic event refers to an emotional experience, such as extreme fear or helplessness, when directly witnessing an occurrence that threatens the physical well-being of an individual [7].

In this study, traumatic event experiences were measured using the traumatic 11 items developed by Cho [31]. Participants were asked how often they have experienced traumatic events such as “unexpected death of a patient” or “unable to resuscitate the patient despite continuous treatment” while caring for COVID-19 patients over the past year (1 = experienced rarely to 5 = experienced very often). The higher the score, the greater the traumatic event experience. In the study by Cho [31], the reliability of the tool was Cronbach’s α = 0.80; in this study, it was 0.804.

#### 2.2.3. Post-Traumatic Stress Disorder Symptoms

PTSD is a disorder that occurs following a severe traumatic event [7].

This study measured PTSD symptoms using the Post-traumatic Diagnosis Scale (PDS) developed by Foa, Cashman, and Perry [32] and was translated and validated in Korean by Nam et al. [33].

The PDS consists of 17 items on PTSD symptoms and three domains: “reexperiencing” (5 items), “avoidance” (7 items), and “arousal” (5 items). Participants were asked on a weekly basis how often they had experienced each symptom in the past month (0 = *not at all* to 3 = *5 or more times a week*).

According to Ahn [34], a total score of 10 points or less was classified as mild, 11–20 points as moderate, and 21 points or more as severe.

In the study by Foa et al. [32] the reliability of the tool was Cronbach’s α = 0.91; in the study by Nam et al. [33], it was 0.95; and in this study, it was 0.936.

#### 2.2.4. Self-Disclosure

Self-disclosure refers to the level of self-exposure that an individual who has experienced a traumatic event provides verbally about the event [14]. This study measured self-disclosure using the self-disclosure scale developed by Han et al. [35]. This scale reminds individuals of the most stressful events they have experienced over the past year and asks them about how much they have talked openly about the incident using a seven-point scale. The level of exposure to others and the degree of openness, depth, and frequency of the traumatic event experienced by the person were measured on a 9-item, 7-point Likert scale (1 = *not at all* to 7 = *very much*). The higher the score, the higher the tendency toward self-disclosure. In a study by Han et al. [35], Cronbach’s α = 0.95; in this study, it was 0.956.

#### 2.2.5. Social Support

Social support refers to the act of making people believe that they are cared for and loved and making them proud to know that they are members of an organization that values communication and responsibility [36]. This study used the 12-item tool developed by Caplan [37] and was translated and validated in Korean by Choi and Jo [38]. This instrument consists of support from supervisors (6 items) and colleagues (6 items) and is measured using a 5-point Likert scale (1 = not at all to 5 = strongly agree); the higher the score, the greater the social support. The reliability of the tool was Cronbach’s α = 0.89 in the study by Choi et al. [38]; in this study, it was 0.930 and 0.934.

#### 2.2.6. Rumination

Rumination refers to invasive reflection in which images or thoughts about a traumatic event automatically emerge and intentional reflection that recalls why such an event occurred and its meaning [13].

In this study, we used the Event-Related Rumination Inventory developed by Cann et al. [39] and translated and validated in Korean by Ahn et al. [40]. This instrument consists of 20 items across two domains, with 10 items each for intrusive rumination and 10 items for deliberate rumination. Items are rated using a 4-point Likert scale (0 = never done to 3 = frequently), with higher scores indicating greater use of a ruminant pattern in that domain. The reliability of the tool was Cronbach’s α =0.96 for intrusive and 0.95 for deliberate rumination in the study by Ahn et al. [40]; in this study, it was 0.955 and 0.954, respectively.

#### 2.2.7. Meaning in Life

The meaning in life is the realization of the true nature of human existence [41]. This study measured meaning in life using the Meaning in Life Questionnaire developed by Steger et al. [42] and translated and validated in Korean by Won et al. [43]. This instrument consists of ten items across two domains with five items for finding meaning and five items for seeking meaning. Items are rated on a 7-point Likert scale (1 = not at all to 7 = always). The reliability of the tool was Cronbach’s α = 0.82 for finding meaning and 0.87 for seeking meaning in the study of Won et al. [43]; in this study, it was 0.843 and 0.926, respectively.

#### 2.2.8. Resilience

Resilience refers to psychological and social characteristics that enable individuals to overcome, adapt, and grow even in the face of serious life challenges, such as hardship or adversity [44]. This study used the Korean translation (K-CD-RISC) [45] of the Connor–Davidson Resilience Scale (CD-RISC) [44]. The tool consists of 25 items rated on a 5-point Likert scale, with nine items on hardness, eight items on persistence, four items on optimism, two items on support, and two items on spirituality in nature. Items were scored on a 5-point Likert scale (0 = not at all to 4 = strongly agree), with higher scores indicating higher resilience. In the study by Baek et al. [45], Cronbach’s α = 0.93; in this study, it was 0.952.

### 2.3. Data Collection

The purpose and protocol of this study were explained to the nursing departments of three hospitals designated for COVID-19 in Seoul, Republic of Korea. Informed consent was obtained for the data collection. The surveys were distributed to nurses who voluntarily agreed to participate in the study and were delivered through the head nurse of their department. The data collection period was from 1 May to 31 July 2021.

### 2.4. Statistical Analysis

The collected data were analyzed using the IBM SPSS 25 (IBM Inc., Armonk, NY, USA). The participants’ general characteristics and research variables were calculated as frequencies, percentages, means, and standard deviations. The differences in PTG according to participants’ general characteristics were analyzed using independent t-tests, ANOVA, and Scheffé tests for the post-test. The correlation between the study variables was calculated using Pearson’s correlation coefficients, and hierarchical multiple regression was performed in three steps to confirm the effect on participants’ PTG.

General characteristics showing a significant difference in PTG were input in the first stage, and factors constituting the PTG model were input in the second stage. In the third stage, the factors that were found to affect PTG in previous studies and the variables of meaning in life and resilience were added.

### 2.5. Ethical Considerations

This study was conducted with the permission and cooperation of the nursing departments of three COVID-19 hospitals in Seoul and after receiving approval from the Institutional Review Board of the Seoul Medical Center in Korea (SEOUL 2021-04-006). Study participants were individually provided with the research guide and consent form along with the questionnaire. They were asked to complete a written consent form if they agreed to participate in the study. The questionnaires were encoded and processed to protect the participants’ personal information.

## 3. Results

### 3.1. Participants’ Characteristics and Differences in Post-Traumatic Growth

Table 1 presents the participants’ general characteristics. The average age was 32.08 (±7.24) years, and those under the age of 29 accounted for the majority (48.1%). By sex, 92.7% of the participants were female. In total, 69.5% were single and 30% lived alone. As for the level of education, 76.4% had a bachelor’s degree, and 67.8% had no religion. The mean number of years of experience was 7.88 (±6.85). Regarding COVID-19 infection, 19.4% experienced the infection themselves or witnessed it in their families, relatives, colleagues, neighbors, etc., and 15.5% experienced self-quarantine. The participants worked in protective equipment for an average of 171.95 (±81.17) days.

The degree of participants’ PTG differed significantly by age group (F = 5.355, *p* = 0.001), marital status (t = 4.980, *p* < 0.001), and religious status (t = 4.204, *p* < 0.001). The degree of PTG was significantly higher in those in their 50 s than in those in their 20 s; it was also significantly higher in married and religious people (see Table 1).

### 3.2. Descriptive Statistics of the Research Variables

The average total score for PTG was 60.15 ± 24.59, the average total score for traumatic event experience was 20.85 ± 5.32, and the average total score for self-exposure was 32.10 ± 10.93. Social support was high with a score of 45.75 ± 6.50, while the average total rumination score was 15.26 ± 12.85, with invasive rumination at 7.14 ± 6.87 and intentional rumination at 8.12 ± 7.10. Meaning in life was found to be high with an average of 46.13 ± 10.03, and resilience was average with 56.86 ± 15.06 points. The mean total score of PTSD was 6.48 ± 7.52, indicating a low trend; although 6% of the participants had severe PTSD and 18.04% had moderate PTSD (see Table 2).

### 3.3. Correlation between the Research Variables

Participants’ PTG rate was measured by meaning in life (r = 0.555, *p* < 0.001), resilience (r = 0.490, *p* < 0.001), and intentional rumination (r = 0.381, *p* < 0.001). Self-exposure (r = 0.227, *p* < 0.001), traumatic event experience (r = 0.212, *p* = 0.001), invasive rumination (r = 0.185, *p* = 0.005), and social support (r = 0.172, *p* = 0.008) were significantly and positively correlated. However, there was no significant correlation with PTSD (see Table 3).

### 3.4. Factors Affecting Participants’ Post-Traumatic Growth

In the first step, an independent variable that was found to have statistically significant differences in PTG among general characteristics according to age, marital status, and religious status were entered to identify the factors affecting participants’ PTG.

In the second stage, traumatic event experience, self-exposure, social support, invasive reflection, and intentional reflection, which constitute the PTG model, were analyzed.

In the third stage, the final analysis was conducted by introducing meaning in life and resilience, which are factors known to affect PTG in previous studies, which this research aimed to add and verify.

As a result of testing the basic assumption of the regression analysis, the correlation between the independent variables was −0.219~0.60, confirming that they were independent of each other. The tolerance was 0.437 to 0.969, which was more than 0.1, and the variance inflation factor was 1.073–2.288, which was less than 10, indicating that no variables had a multicollinearity problem. The residuals were analyzed, and normality and equal variance of the error term and linearity of the model were confirmed. In the autocorrelation test, the Durbin–Watson statistic was 2.090, which is close to 2, confirming the absence of autocorrelation.

Hierarchical regression results for the PTG change of the determined variables in Model 1 included only general characteristics, which were the control variables. Marital status (β = 0.208, *p* = 0.014) and religious status (β = 0.221, *p* < 0.001) were statistically significant variables; the explanatory power of the three variables was 12.6% (F = 12.130, *p* < 0.001). Married and religious participants also had higher PTG rates (Table 4).

In Model 2, self-disclosure (β = 0.152, *p* = 0.008), social support (β = 0.172, *p* = 0.003), and intentional rumination (β = 0.393, *p* < 0.001) were the most influential predictive variables, and the explanatory power of the five variables was 18.4% (*p* < 0.001), resulting in a total explanatory power of 29.7% (F = 13.249, *p* < 0.001).

In Model 3, meaning in life (β = 0.294, *p* < 0.001) and resilience (β = 0.207, *p* = 0.001) were predictors of this effect, and the explanatory power of these two variables was 16.2% (*p* < 0.001). The total explanatory power was 46.0% (F = 20.758, *p* < 0.001) (see Table 4).

## 4. Discussion

This study aimed to identify the factors related to PTG among nurses at hospitals dedicated to COVID-19 in Korea. The results indicated that if they were married or religious, the older the participant, the higher the PTG score. These results are similar to those of non-COVID-19 hospitals in the province [46]. Therefore, this study is meaningful in that it more clearly identifies the PTG factors of nurses in pre-COVID-19 hospitals in Korea.

Factors related to PTG such as age, marriage, and religion were identified for nurses in charge of COVID-19 care in China and Hong Kong [47,48]. However, religion was not considered a related variable in Hong Kong, and there was little statistical relationship between PTG and religion in China. Therefore, the results of this study were somewhat different. In the case of China, the ratio of non-religious or Chinese folk beliefs is more than 70%, whereas only 7% of the total population in Korea do not have religious or folk beliefs [49]. Similarly, a Pakistani paper examining the relationship between PTG and religious beliefs of medical staff dealing with patients infected with coronavirus, including doctors and nurses, also found that religiosity was a positive factor in PTG [50]. Private beliefs are reported to be less than 3% [49], so it is thought that similar results to this study were derived when there were many coronavirus specialists with religious beliefs.

In this study, 24.0% (56) of the nurses had moderate PTSD or higher, but the overall average was low. This might be because 28.8% (67) of the participants had less than three years of experience. When looking at the impact of the COVID-19 pandemic on health care workers’ (doctors and nurses) mental health, previous studies indicated approximately 38.8% of all healthcare workers were affected. Among them, 42.3% of nurses had symptoms of PTSD [51].

However, the Korean government designated dedicated hospitals for COVID-19 patients, and intensively deployed experienced nurses at the beginning of the pandemic. As a result, the ratio of beginner to novice nurses was relatively small; accordingly, it was judged that the percentage of PTSD was also small. Therefore, in the future, highly skilled and experienced nurses, instead of inexperienced ones, should be assigned as nursing personnel in hospitals dedicated to new infectious diseases, such as COVID-19. This may help minimize the trauma experienced by the staff.

The participants in this study had moderate experiences of traumatic events, self-exposure, and post-traumatic growth and resilience. Invasive and intentional rumination were low, and social support and meaning in life were high. As the COVID-19 situation has continued for a long time, this may be because the experience or recovery from the incident has progressed, to some extent, beyond rumination to an understanding of the incident, and there is urgency regarding coping with the situation and long-term fatigue. However, it is possible that the positive social atmosphere for medical staff in charge of COVID-19, such as the public support campaign in Korea, contributed to their social support and sense of meaning in life. The campaign is named “Thanks to Challenge,” and the public has conducted it by posting sign language gestures that mean respect and pride on social networking sites [52] to thank the medical staff for their hard work and dedication. The nurses in charge of COVID-19 care are believed to have felt that this social support increased their sense of meaning in life through the campaign.

The degree of growth after trauma showed a significant positive correlation with meaning in life, resilience, intentional rumination, self-exposure, traumatic event experience, invasive rumination, and social support. These results are consistent with those of previous studies that showed a significant correlation between growth and resilience and the meaning in life after trauma of medical staff [53] and are similar to those of COVID-19 survivors [54]. Therefore, most of the PTG-related factors suggested in the conceptual framework of this study have been identified. However, PTG did not show a significant association with PTSD, which is meaningful in that the former is correlated with the experience of traumatic events, but not necessarily related to the latter. In other words, a veteran nurse may experience a severe traumatic event and not develop a disability, but experiencing that traumatic event can still lead to PTG.

Marital status, religion, self-exposure, deliberate rumination, meaning in life, and resilience were significant factors affecting PTG. Meanwhile, age, traumatic event experience, social support, and intrusive rumination were not significant. Intrusive rumination is the repeated recall of an event that increases psychological distress, but paradoxically, provokes intentional rumination [39]. A previous study confirmed that intrusive rumination and deliberate rumination have a double mediating effect on PTG [55]. However, the present study identified that intentional rumination as a voluntary cognitive process in understanding such an experience had a more positive effect on PTG than continuously recalling the situation of COVID-19 (invasive rumination). This is because COVID-19 has been ongoing since 2019. Instead of being immersed in a situation, intentional rumination is a process of understanding one’s experiences and finding a positive meaning in life. Accordingly, as new infectious diseases are likely to appear in the future, it is important to secure policy support, such as the Nursing Act, which protects nurses’ rights and interests and provides direct compensation beyond temporary campaigns.

## 5. Conclusions

This study investigated the post-traumatic stress experienced by nurses working at a designated hospital during the COVID-19 pandemic and the PTG factors that helped to overcome that stress. Through this, it was possible to confirm the theoretical framework of factors influencing PTG. This study identified marital status, religious status, self-exposure, intentional rumination, resilience, and meaning in life as factors affecting the PTG of nurses in charge of COVID-19 care. Based on this, a program can be planned to promote post-traumatic growth by drawing on meaning in life, social support, and rumination.

Concurrently, this study has the following limitations. COVID-19 is a new infectious disease, and nurses’ experiences and responses when it first broke out are different from those in the current situation, with nurses having had two years of experience with the crisis. Therefore, it is possible that there are differences in nurses’ growth factors after trauma when they first encounter a new infectious disease. In addition, due to the continuing COVID-19 situation, nurses’ fatigue may be aggravated and the intensity of the situation may be dulled, which may have lowered their awareness of the current situation.

In addition, this study was conducted with nurses in a few selected hospitals dedicated to COVID-19 in Korea. Therefore, it is proposed to conduct repeated research not only for hospital nurses, but also for nurses at nursing hospitals and nursing homes, school nurses, and nurses at screening clinics who are experiencing post-traumatic stress due to COVID-19.

In sum, when new infectious diseases are emerging, personal reflection and social and institutional support are required to strengthen nurses’ resilience and their sense of meaning in life to promote PTG.

## Figures and Tables

**Table 1:** Differences in Post-Traumatic Growth according to Participants’ Characteristics (N = 233).

Characteristics	Categories	N (%) or M ± SD		Post-Traumatic Growth			
				M ± SD	t/F	*p*	Scheffé
Age (year)	(range: 23–57)	32.08 ± 7.24					
	≤29 ^a^	112	48.1	2.17 ± 0.97	5.355	0.001	a < d
	30–39 ^b^	84	36.1	2.54 ± 1.02			
	40–49 ^c^	31	13.3	2.74 ± 0.76			
	≥50 ^d^	6	2.6	3.17 ± 0.34			
Gender	Female	216	92.7	2.42 ± 0.98	0.048	0.962	
	Male	17	7.3	2.41 ± 1.04			
Marital status	Married	71	30.5	2.85 ± 0.82	4.980	<0.001	
	Not married	162	69.5	2.23 ± 0.99			
Cohabitation	Live alone	70	30.0	2.26 ± 1.03	−1.524	0.129	
	Do not live alone	163	70.0	2.47 ± 0.96			
Educational background	Associate degree	47	20.2	2.28 ± 0.99	2.455	0.088	
	Bachelor degree	178	76.4	2.41 ± 0.99			
	Master degree	8	3.4	3.11 ± 0.49			
Religion	Have	75	32.2	2.80 ± 0.96	4.204	<0.001	
	Do not have	158	67.8	2.24 ± 0.94			
Total career (year)	(range: 0.5–35.58)	7.88 ± 6.85					
	≤1 (Beginner)	18	7.73	2.44 ± 1.06	2.140	0.062	
	3 (Novice)	49	21.03	2.27 ± 1.00			
	5	42	18.03	2.10 ± 1.11			
	10	58	24.89	2.44 ± 0.90			
	20	54	23.18	2.62 ± 0.90			
	>20	12	5.15	2.88 ± 0.82			
Self-quarantine experience	Yes	36	15.5	2.63 ± 0.94	1.368	0.173	
	No	197	84.5	2.39 ± 0.99			
Duration of work wearing D-level ^†^ (day)	(range: 2–395)	171.95 ± 81.17			1.619	0.186	
	≤50	21	9.0	2.77 ± 0.87			
	51–150	74	11.6	2.27 ± 1.01			
	151–250	86	20.2	2.37 ± 0.96			
	>251	39	27.0	2.52 ± 1.03			
PTSD ^‡^	Mild (1–10)	177	76.0	2.36 ± 0.98	1.415	0.245	
	Moderate (11–20)	42	18.0	2.63 ± 0.99			
	Severe (more than 21)	14	6.0	2.27 ± 1.00			

^†^ excluded missing value, ^‡^ PTSD: post-traumatic stress disorder, ^a^ age ≤ 29, ^b^ age 30–39, ^c^ age 40–49, ^d^ age ≥ 50.

**Table 2:** Level of the research variables (N = 233).

Variables	Items (Score)	Range	Total M ± SD	Item M ± SD
Post-traumatic growth	25 (0–5)	5–114	60.15 ± 24.59	2.41 ± 0.98
Changed perception of self	8 (0–5)	0–38	20.78 ± 8.62	2.60 ± 1.08
Relation to others	5 (0–5)	0–24	12.39 ± 5.39	2.48 ± 1.08
Spiritual-existential change	7 (0–5)	0–34	13.36 ± 7.81	1.91 ± 1.12
New possibilities	5 (0–5)	0–25	13.62 ± 4.73	2.72 ± 0.95
Trauma events experiences	11 (1–5)	11–38	20.85 ± 5.32	1.90 ± 0.48
Self-disclosure	9 (1–7)	9–63	32.10 ± 10.93	3.57 ± 1.21
Social support	12 (1–5)	27–60	45.75 ± 6.50	3.81 ± 0.54
Rumination	20 (0–3)	0–60	15.26 ± 12.85	0.76 ± 0.64
Intrusive rumination	10 (0–3)	0–30	7.14 ± 6.87	0.71 ± 0.69
Deliberate rumination	10 (0–3)	0–30	8.12 ± 7.10	0.81 ± 0.71
Meaning in life	10 (1–7)	13–70	46.13 ± 10.03	4.61 ± 1.00
Presence of meaning	5 (1–7)	8–35	22.42 ± 5.37	4.48 ± 1.07
Search for meaning	5 (1–7)	5–35	23.72 ± 5.56	4.74 ± 1.11
Resilience	25 (0–4)	21–100	56.86 ± 15.06	2.27 ± 0.60
PTSD ^†^	17(0–3)	0–34	6.48 ± 7.52	0.38 ± 0.44

^†^ PTSD: post-traumatic stress disorder.

**Table 3:** Correlation between the research variables (N = 233).

Variables	1	2	3	4	5	6	7	8	9
	r (*p*)	r (*p*)	r (*p*)	r (*p*)	r (*p*)	r (*p*)	r (*p*)	r (*p*)	r (*p*)
1. Post-traumatic growth	1	0	0	0	0	0	0	0	0
2. Trauma events	0.212 (0.001)	1	0	0	0	0	0	0	0
3. Self-disclosure	0.227 (<0.001)	0.174 (0.008)	1	0	0	0	0	0	0
4. Social support	0.172 (0.008)	0.145 (0.027)	0.092 (0.161)	1	0	0	0	0	0
5. Intrusive rumination	0.185 (0.005)	0.245 (0 < 0.001)	0.159 (0.015)	−0.159 (0.015)	1	0	0	0	0
6. Deliberate rumination	0.381 (<0.001)	0.290 (<0.001)	0.171 (0.009)	−0.095 (0.146)	0.691 (<0.001)	1	0	0	0
7. Meaning in life	0.555 (<0.001)	0.162 (0.013)	0.077 (0.241)	0.160 (0.015)	0.050 (0.444)	0.287 (<0.001)	10	0	0
8. Resilience	=0.490 (<0.001)	0.138 (0.035)	0.052 (0.432)	0.206 (0.002)	0.004 (0.947)	0.176 (0.007)	0.600 (<0.001)	1	0
9. PTSD ^†^	0.030 (0.649)	0.239 (<0.001)	0.080 (0.222)	−0.219 (0.001)	0.528 (<0.001)	0.375 (<0.001)	−0.170 (0.009)	−0.167 (0.011)	1

^†^ PTSD: post-traumatic stress disorder.

**Table 4:** Factors Affecting Post-Traumatic Growth (N = 233).

Variables	Model 1			Model 2			Model 3					
	B ^‡^	β ^§^	*p*	B ^‡^	β ^§^	*p*	B ^‡^	SE ^∥^	β ^§^	t	*p*	VIF *
(constant)	43.935	0	0	−3.648	0	0.790	−33.804	12.578	0	−2.688	0.008	0
Age (yr)	0.229	0.070	0.403	0.146	0.045	0.568	0.154	0.223	0.047	0.691	0.491	2.023
Marriage ^†^	10.617	0.208	0.014	10.138	0.199	0.009	8.099	3.391	0.159	2.388	0.018	1.898
Religion ^†^	11.129	0.221	0.000	9.282	0.185	0.001	5.761	2.535	0.115	2.273	0.024	1.092
Trauma events	0	0	0	0.200	0.045	0.448	0.081	0.231	0.018	0.353	0.725	1.171
Self-disclosure	0	0	0	0.327	0.152	0.008	0.320	0.108	0.149	2.974	0.003	1.073
Social support	0	0	0	0.622	0.172	0.003	0.329	0.187	0.091	1.759	0.080	1.146
Intrusive rumination	0	0	0	−0.377	−0.110	0.158	0.000	0.238	0.000	−0.001	0.999	2.065
Deliberate rumination	0	0	0	1.301	0.393	0.000	0.673	0.242	0.203	2.785	0.006	2.288
Meaning in life	0	0	0	0	0	0	0.689	0.149	0.294	4.632	0.000	1.725
Resilience	0	0	0	0	0	0	0.323	0.096	0.207	3.377	0.001	1.607
R^2^	0.137			0.321			0.483					
Adj. R^2^	0.126			0.297			0.460					
⊿Adj. R^2^	0.137			0.184			0.162					
F (*p*)	12.130 (<0.001)			13.249 (<0.001)			20.758 (<0.001)					
⊿ F (*p*)	12.130 (<0.001)			12.149 (<0.001)			34.800 (<0.001)					

^†^ dummy variable (0 = No, 1 = Yes), B ^‡^ = unstandardized coefficients, β ^§^ = standardized coefficients, SE **^∥^** = standard error, VIF * = variance inflation factor.
